# Positive Effects of Aerobic-Resistance Exercise and an Ad Libitum High-Protein, Low-Glycemic Index Diet on Irisin, Omentin, and Dyslipidemia in Men with Abdominal Obesity: A Randomized Controlled Trial

**DOI:** 10.3390/nu16203480

**Published:** 2024-10-14

**Authors:** Agnieszka Suder, Karol Makiel, Aneta Targosz, Piotr Kosowski, Robert M. Malina

**Affiliations:** 1 Department of Anatomy, Faculty of Physical Rehabilitation, University of Physical Education, 31-571 Cracow, Poland; 2 Department of Physiology, Faculty of Medicine, Jagiellonian University Medical College, 31-531 Cracow, Poland; 3 Department of Petroleum Engineering, AGH University, 30-059 Cracow, Poland; 4 Department of Kinesiology and Health Education, University of Texas at Austin, Austin, TX 78712, USA; 5 School of Public Health and Information Sciences, University of Louisville, Louisville, KY 40202, USA

**Keywords:** aerobic-resistance exercise, abdominal obesity, body mass index, irisin, omentin, low-glycemic index, carbohydrates, fiber, protein intake

## Abstract

Objectives: The aim of this research was to evaluate changes in body composition, adipokine levels, and dyslipidemia parameters in males with abdominal obesity following two distinct interventions: exercise alone and exercise combined with an ad libitum diet. Methods: This study included 44 males with abdominal obesity (mean age 34.7 ± 5.5 years, waist circumference [WC] 110.3 ± 8.5, BMI 32.0 ± 3.9), who were randomly assigned to three groups: an experimental group engaging in aerobic-resistance exercise (II, *n* = 16), an experimental group engaging in aerobic-resistance exercise combined with an ad libitum high-protein, low-glycemic index carbohydrate diet (III, *n* = 16), both interventions lasting 6 weeks, and a control group without interventions (I, *n* = 12). Body composition metrics (body mass index [BMI], waist circumference [WC], body fat [BF], abdominal fat [ABD]) and fat-free mass [FFM], along with biochemical blood analyses (irisin [IR], omentin [OMEN], glucose [GLU], insulin [INS], LDL- and HDL-cholesterol), were measured at baseline and after the 6-week intervention. The effects of the interventions on the analyzed variables across groups were assessed using mixed ANOVA tests with post-hoc comparisons. Effect size (ES) was also calculated using partial eta squared (*η*p^2^). Results: The intervention in group III resulted in a significant decrease in IR (*p* < 0.01, *η*p^2^ = 0.03) by 41% and LDL-C (*p* < 0.01, *η*p^2^ = 0.02) by 14%. These effects were associated with a reduction in BF (*p* < 0.01, *η*p^2^ = 0.02) by 14%, ABD (*p* < 0.01, *η*p^2^ = 0.03) by 31%, and WC (*p* < 0.01, *η*p^2^ = 0.01) by 3%. In group II, decreases after 6 weeks of intervention were noted only in WC (*p* = 0.02, *η*p^2^ = 0.01) by 1% and in INS (*p* < 0.01, *η*p^2^ = 0.04) by 47%. No differences were found between groups. The use of low-glycemic index carbohydrates (*p* < 0.01, *η*p^2^ = 0.06) and increased protein intake (*p* < 0.01, *η*p^2^ = 0.30) led to changes in the fiber-to-energy value of the diet ratio (*p* < 0.01, *η*p^2^ = 0.18) and a reduction in dietary energy value (*p* < 0.01, *η*p^2^ = 0.13) by 23%, resulting in a greater energy deficit than in the II group. Conclusions: These findings highlight the effect of combining dietary and exercise interventions to achieve significant changes in body composition and metabolic parameters, even over a short period of intervention.

## 1. Introduction

Obesity is a chronic condition driven by an excess of dietary energy and reduced energy expenditure associated with chronically low physical activity levels [1]. Lifestyle interventions aimed at reducing dietary energy intake and increasing energy expenditure have limited effectiveness associated with complex and persistent hormonal, psychological, metabolic, and neurochemical adaptations that promote weight regain [1,2]. Managing the treatment process for obesity, which often involves pharmacological treatment, psychotherapy, and bariatric surgery, also aims to modify dietary habits and select specialized exercises to achieve long-term effects [3,4].

The Obesity Medicine Association (OMA) Clinical Practice Statement (CPS) on Nutrition and Physical Activity in the care of patients with excessive adipose tissue and adiposopathic metabolic consequences recommends increasing the intake of vegetables, leafy greens, fruits, berries, legumes, whole grains, complex carbohydrates, high-fiber foods, minimally processed and whole foods, and using sweeteners such as aspartame or sucralose while limiting simple sugars, low-fiber foods, ultra-processed, packaged foods, energy-dense foods, and beverages [5]. The main goal of these recommendations is to reduce the high glycemic index of the diet while maintaining an adequate intake of slowly digestible carbohydrates. Another recommendation for qualitative dietary changes is to increase the amount of high-quality protein, which leads to a reduction in fat mass, helps to maintain a high level of lean mass, and stimulates the satiety center [6].

Implementing dietary interventions focused on qualitative changes rather than quantitative aspects of the diet is also justified by psychological mechanisms. Individuals who overly focus on controlling the energy content of their diet and maintaining a predetermined nutrient ratio tend to unconsciously compensate by overeating because of disrupted self-regulation processes [7]. Permitting a choice among a wide range of products that align with the goals of the interventional diet can function to reduce the need for strict control over meal caloric content and nutrient composition and, in turn, improve prospects for the long-term maintenance of healthy eating practices.

Exercise training is an effective tool in treating obesity, including abdominal obesity [8,9]. When preparing exercise training protocols for weight loss in patients with abdominal obesity, it is important to consider the risk of reducing the basal metabolic rate [1], which may be a consequence of the physiological disturbances associated with obesity. The use of exercise aimed at increasing energy expenditure and improving health should not only lead to a reduction in fat mass but also to skeletal muscle hypertrophy. Skeletal muscles, through high levels of thermogenesis, can help maintain an adequate basal metabolic rate [10]. To achieve the intended goals, it is necessary to appropriately combine the type and intensity of exercise [11]. Increasingly, resistance exercise is recommended amongst lifestyle changes in patients with obesity in order to increase muscle mass and reduce the risk of metabolic diseases [12,13]. Moreover, combining resistance exercise with aerobic training can function to reduce abdominal obesity, insulin resistance, and adipokine levels than aerobic exercise alone [14,15,16]. The implementation of physical exercise and dietary interventions affect, among others, the level of IR (irisin) and OMEN (omentin), adipokines, which are significantly affected by abdominal obesity.

IR is both an adipokine and a myokine, primarily produced in skeletal muscles and, to a lesser extent, in adipose tissue in response to physical exercise [17]. IR is responsible for morphological changes in adipose tissue, an increase in the body’s total energy expenditure, improvement in cellular insulin sensitivity, and reduction in lipid disorders associated with obesity [18,19]. Resistance training has a more significant impact on IR production compared with moderate-intensity or high-intensity interval training [20]. The diet also influences IR synthesis in the bloodstream [21]. These relationships are important for both healthy individuals and patients with abdominal obesity, who are at risk of IR resistance [20,22].

OMEN is an adipokine synthesized mainly in abdominal adipose tissue [23]. Decreased levels of OMEN are observed in obese individuals, and their reduction leads to metabolic disorders such as insulin resistance and glucose intolerance [24]. The secretion of OMEN-1 is stimulated by physical activity and a diet with a negative energy balance [25,26].

The objective of our research was to evaluate the impact of a 6-week aerobic-resistance exercise per se and an aerobic-resistance exercise combined with an ad libitum high-protein, low-glycemic index diet on the concentrations of irisin (IR) and omentin (OMEN), as well as on cholesterol levels in men with abdominal obesity, compared with men with abdominal obesity who did not participate in any intervention (control group). Changes in body composition and selected metabolic indicators were tracked.

Accordingly, it was hypothesized that aerobic-resistance exercise combined with a high-protein, low-glycemic index diet would be associated with more favorable changes in adipokine levels, reduced markers of atherogenic dyslipidemia, and improved body composition compared with aerobic resistance exercise alone in men with abdominal obesity.

## 2. Materials and Methods

### 2.1. Study Design

This research utilized a prospective, randomized, and controlled study framework.

The variables analyzed in this manuscript are part of a larger research project in which we investigated the effect of exercises and dietary intervention on body composition, as well as biochemical and hormonal parameters in men with abdominal obesity.

In addition to being male and having abdominal obesity, this study included the following criteria: age between 30 and 40 years; written consent for voluntary participation in the research; a medical certificate confirming no contraindications for aerobic-resistance exercises; the absence of health contraindications such as ischemia, decompensated heart failure, heart rhythm disorders, severe pulmonary hypertension, symptomatic aortic stenosis, acute myocarditis, endocarditis or pericarditis, uncontrolled blood pressure, aortic dissection, Marfan syndrome, uncontrolled diabetes, mental disorders, health issues preventing movement. Participation in any other physical activity program during the course of the project was also prohibited. However, they were permitted to engage in physical activities associated with their daily routines.

Exclusion criteria for the research project included the lack of a medical certificate confirming the ability to engage in resistance and aerobic training and insufficient attendance at training sessions in the intervention groups (minimum attendance was set at above 90%).

Participants (*n* = 53) were randomly allocated to one of the three groups through a process that involved selecting an opaque envelope containing a number assigned to each group. The assignment to the intervention groups was managed by a trainer. The recruitment and intervention phase lasted from February 2022 to July 2022, ensuring that the study groups met the minimum sample size requirements.

**Figure 1:**
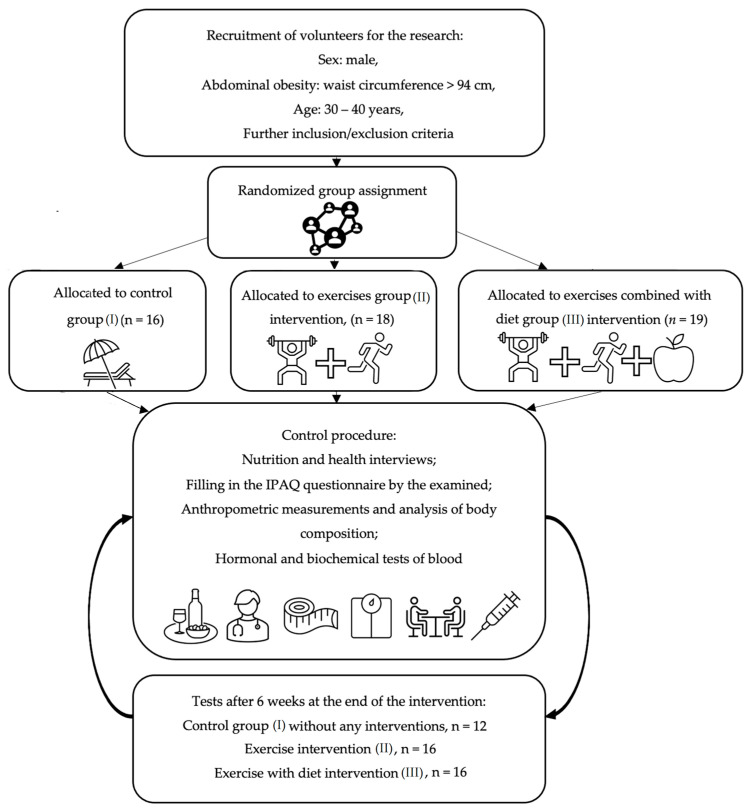
Study flowchart.

The volunteers (*n* = 53) received a written description of the goals, procedures, and planned course of the research project. All participants provided written consent for the processing of personal data, voluntary participation in this study, and the use of obtained results for scientific purposes. Informed consent was obtained from all subjects. Each participant could withdraw from this study at any time without facing any consequences. Instances of patient resignation from the project and participant exclusion were recorded and were due to the following reasons: missing more than 10% of training sessions (2 cases), non-compliance with dietary recommendations (3 cases), uncontrolled alcohol consumption (1 case), and absence from control sessions (3 cases). During the study, 4 participants from group I, 2 participants from group II, and 3 participants from group III withdrew, and the final number of analyzed participants was n = 44. The flowchart of this study is presented in Figure 1.

There were no occurrences of harm or adverse events reported during the trial period. Given the nature of the interventions or the lack of an intervention, a blinding procedure was not applied. However, to minimize potential biases, the laboratory staff, the biostatistician, and the team assessing outcomes were kept unaware of the group assignments.

This research project was approved by the Ethics Committee of the Regional Chamber of Physicians in Krakow (15/KBL/OIL/2022), and all methods adhered to the guidelines of the Declaration of Helsinki. This study was also registered in the Australian New Zealand Clinical Trials Registry under the registration number ACTRN12624000184572 (26 February 2024), following CONSORT guidelines.

### 2.2. Sample

This study involved 44 Caucasian men 30 to 40 years (mean 34.7 ± 5.5 years) who met the primary selection criterion for abdominal obesity, defined as a waist circumference (WC) greater than 94 cm [27]. The project participants were working individuals from the community of a large residential estate in the city of Cracow recruited via social media. The participants were randomly divided into three groups:

Control group (I)—12 men (34.1 ± 5.5 years, body mass BM 101.9 ± 11.5 kg, waist circumference (WC) 111.2 ± 6.8) who were familiar with the recommendations for the treatment of obesity but did not undergo any intervention;

Experimental group (II)—16 men (34.8 ± 6.0 years, BM 105.6 ± 7.4 kg, WC 110.8 ± 11.3) who performed aerobic-resistance exercises;

Experimental group (III)—16 men (34.9 ± 5.6 years, BM 105.6 ± 10.3 kg, WC 109.0 ± 7.6) who participated in aerobic-resistance exercises and had an ad libitum high-protein, low-glycemic index diet.

The baseline characteristics of the participants in the three groups are summarized in Table 1. The groups did not significantly differ in age and basic anthropometric measurements at baseline.

### 2.3. Methods

Assessments were conducted for all participants at two points in time, before and after 6 weeks of the intervention:

#### 2.3.1. Anthropometry

Height (Ht, cm) was measured to the nearest millimeter in a standing position without shoes, with the head aligned in the Frankfurt plane, using a stadiometer (Seca 231 stadiometer, Hamburg, Germany). Body mass (BM, kg) was measured in a standing position using a standardized medical scale (Beurer PS 240, Budapest, Hungary) with an accuracy of 50 g. Waist circumference (WC, cm) was measured to the nearest millimeter using an anthropometric tape between the lower edge of the ribcage and the upper edge of the iliac crest, with the participant standing and measurements taken at the end of a gentle expiration. The body mass index (BMI, kg/m^2^) was calculated.

#### 2.3.2. Body Composition

Dual-energy X-ray Absorptiometry (DEXA) was used to estimate body composition: body fat (BF, kg), abdominal fat (ABD, kg), and fat-free mass (FFM, kg). A Lunar Prodigy Primo PR+352163 (Chicago, IL, USA) was used.

#### 2.3.3. Adipokines

Fasting blood samples were collected in the morning following a 24 h break from training, from the basilic, cephalic, or median cubital vein into test tubes (Vacumed^®^ system, F.L. Medical, Torreglia, Italy) by an experienced nursing team. The blood was centrifuged (RCF 1000× *g*) immediately after collection for 15 min at 4 °C (MPW-351R, MPW Med. Instruments, Warsaw, Poland), and the serum was collected and stored at −80 °C until further analysis (BIO Memory 690L, Froilabo, Paris, France). The concentrations of irisin (IR, ng/mL) and omentin (OMEN, ng/mL) were measured using commercially available ELISA kits according to the manufacturer’s protocol. The human IR ELISA Kit (catalog number 201-12-5328) was purchased from Shanghai Sunred Biological Technology Co. (Shanghai, China). The human OMEN ELISA kit (catalog number 201-12-0156) was bought from Shanghai Sunred Biological Technology Co. (Shanghai, China). An ELx 808 spectrophotometric microplate reader (BioTek, Winooski, VT, USA) was used to determine the optical density at 450 nm. These measurements were performed at the Laboratory of Genetics and Molecular Biology, Department of Physiology, Jagiellonian University Medical College, Krakow, Poland.

#### 2.3.4. Biochemical Blood Indices

The glucose (GLU) (mmol/L) concentration in blood plasma was measured using the enzymatic method with a Cobas c701/702 biochemical analyzer (Roche Diagnostics International Ltd., Mannheim, Germany). The serum insulin (INS) (µIU/mL) concentration was estimated with electrochemiluminescence (ECLIA) using the Cobas e801 apparatus (Roche Diagnostics International Ltd., Mannheim, Germany), following the manufacturer’s guidelines using reagents specific to the GLUC3 and Elecsys Insulin analyzers, respectively. Total cholesterol (TC, mmol/L) and high-density lipoprotein cholesterol (HDL-C, mmol/L) were estimated with the spectrophotometric method following the guidelines of the clinical chemistry analyzer Architect ci-4100 (Abbott Laboratories, Wiesbaden, Germany). Intra- and inter-assay coefficients of variation (CV) for the assays were 0.9–1.2% and 1.2–1.8%, respectively. The LDL-C fraction was calculated as follows:

LDL-C (mmol/L) = TC (mmol/L) – HDL-C − (TG (mmol/L)/2.2).

#### 2.3.5. Evaluation of Total Energy Expenditure and Energy Value of Diet

The International Physical Activity Questionnaire (IPAQ) was conducted in three study groups to calculate Non-Exercise Activity Thermogenesis (NEAT). The long IPAQ version was used to assess daily energy expenditure based on self-reported physical activity [28,29]. The estimated energy expenditure during training in the respective intervention groups was measured using a Polar M200 GPS Running Watch with a Wrist-Based Heart Monitor (Kempele, Finland).

To estimate energy intake based on the diets of the participants, a clinical dietitian conducted a 24-h nutrition interview using the nutrition record method. Data from dietary recalls for each time point (baseline, six weeks), two on weekdays and one on a weekend day, were averaged and used to determine overall dietary intake for each time point. Data for each individual were analyzed using the DietaPro program (version 4.0, Institute of Food and Nutrition, Warsaw, Poland) to quantitatively assess nutritional intake and monitor any dietary changes during the intervention and to generate a report detailing dietary nutrients, including proteins (%), carbohydrates (%), fiber (g), and fats (%). Based on the obtained report, the total fiber/energy value of the diet (mJ) index was calculated.

In the group subjected to specific exercises along with dietary intervention, qualitative dietary changes were implemented by increasing protein intake and introducing low glycemic index products (detailed description in Section 2.4.2). Along with a 24-h dietary recall conducted by a dietitian, participants in the intervention groups recorded consumed products daily in the Fitatu dietary program (version 3.41, Fitatu Ltd., Poznan, Poland), which provides a more reliable means of monitoring dietary adherence. For each training session, a clinical dietitian verified the accuracy of data entered into the program and provided consultations related to diet monitoring.

### 2.4. Interventions

#### 2.4.1. Aerobic Resistance Exercises

The exercise interventions for the II and III groups were conducted in a fitness club under the supervision of a personal trainer over a period of six weeks, three times per week, with each session lasting one hour. Training sessions were consistently scheduled in the evening, from 6 to 9 pm, and conducted by the same personal coach. Sessions took place in a controlled environment with a constant temperature of 22 degrees Celsius and maintained humidity of 45%. Adherence to the intervention protocol was tracked through a session attendance checklist. Participants who missed more than 10% of the prescribed training sessions over the 6-week interval were excluded from the analysis.

Before beginning the intervention, participants underwent a one-week training program during which they were instructed by a personal trainer on aerobic-resistance exercises, facilitating initial adaptation to the training regimen. Participants performed a one-repetition maximum (1RM) test, considered the gold standard for assessing muscle strength in non-laboratory situations.

Aerobic-resistance training (see Supplementary Table S1) took place in groups of no more than five individuals. The program began with a five-minute aerobic warm-up on a treadmill at an intensity of 50% HR max. A “push-pull” training approach was applied, alternating engagement of antagonistic muscle groups. On “pull” days, the gluteal muscles, hamstrings, back muscles, rear deltoids, and biceps were trained, while on “push” days, the quadriceps, chest muscles, arm muscles (front and lateral deltoids), and triceps were trained. Each resistance training session included six exercises, four sets per exercise, with 12 repetitions and 60-s breaks between sets. Loads were set at 70% of one-repetition maximum (1RM). An aerobic training component followed the resistance exercises. Participants trained at 70% of maximum heart rate (HR max) on a treadmill (Technogym New Excite Run Now 500, Cesena, Italy), stationary bicycle (Technogym Artis, Cesena, Italy), or elliptical trainer (Precor EFX556i Elliptical, Woodinville, WA, USA). To prevent lower limb overloading, participants alternated between these three devices. The resistance training session lasted 40 min, followed by 10 min of aerobic training. Each session concluded with a five-minute cooldown, including breathing exercises and stretching.

In our previous article on this project, we showed that the interventions combining aerobic and resistance exercise in the II group and the combination of exercise and diet in the III group were associated with a significant increase in energy expenditure (kcal/day) after 6 weeks of intervention compared with the first week of the study [30]. The total energy expenditure in the II group was 2871.9 ± 286.1 kcal/day, and in the III group, it was 2846.7 ± 263.9 kcal/day after 6 weeks of intervention. There was also a significant increase in the loads used during resistance training in the II group by 4.5% and in the III group by 7% (sum of 1RM from 3 exercises: squat, bench press, bar pulldown) after 6 weeks of intervention.

#### 2.4.2. High-Protein, Low-Glycemic Index Carbohydrate Diet

The III group’s dietary regimen focused on high protein intake and included low glycemic index carbohydrates. Sustaining all aspects of such a diet, particularly the correct energy value over an extended period, is highly unlikely; therefore, the primary nutritional education objective was to specify certain food groups. The main energy sources were low glycemic index carbohydrates, including low-energy vegetables and fruits and whole grain products. The second predominant nutritional component was protein, specifically from animal sources such as lean dairy, meat, and fish. The objective of the dietary changes was to achieve an unconscious negative energy balance of approximately 500 kcal per day, resulting in a weekly weight loss of around 0.5 kg and a total of 3 kg over the six weeks of the experiment [31]. The qualitative goal was to achieve high protein intake, approximately 25% of dietary energy, with carbohydrates at 50% and fats at 25%. Low glycemic index carbohydrates are high in fiber; thus, it was assumed that consumption in the III group would exceed 30 g per day.

Due to the dietary intervention and the risks associated with the lack of consistency in maintaining the dietary change assumptions among the study participants, we introduced rigorous control procedures. In addition to regular dietary consultations, participants received individually prepared materials with recipes, a shopping list, essential information, and dietary recommendations. The “shopping center” approach was implemented, as it resembled an ad libitum form of nutritional intervention, allowing individuals the freedom to choose food products within specified parameters [32,33]. A greater feeling of satiety after consuming certain products made it easier to achieve a negative energy balance in this group.

The detailed nutritional intervention included:

- Shopping in designated chain stores with consistent product types and quality (supplied from the same warehouse).
- Participant education using a graphical representation of products for meal preparation. To present high-protein products and low glycemic index carbohydrates, a photo album of food products and dishes was used (Institute of Food and Nutrition, Warsaw, Poland, 2000).
- A personal photo report of nutritional products from a popular shopping center was also used to facilitate shopping and maintain dietary principles.
- Participants used electronic scales, accurate to 1 g, to weigh products before consumption or preparation.
- After each meal, participants recorded consumed products in the Fitatu dietary program (version 3.41, Fitatu Ltd., Poznan, Poland), installed on a mobile application, and received a report on consumed calories and nutrients.
- A qualified dietitian monitored the program report and provided feedback on adherence to dietary parameters.
- If the participant’s diet included products with a high glycemic index or the person consumed too little protein, the dietician recommended changes, proposing products consistent with the project’s assumptions.
- In the event of concerns or questions about the nutritional regime, dieticians were contacted. Eighteen additional individual checks of the subjects’ dietary habits were conducted during the meetings.

### 2.5. Statistical Analysis

The Shapiro–Wilk test was initially used to check for the normal distribution of the sample. As most variables followed a normal distribution, differences between intervention and control groups were analyzed using a two-way mixed-design analysis of variance (ANOVA) for independent groups. The analysis yielded significant effects of group, time, and their interaction. The group effect demonstrates differences in outcomes between the groups (intervention and control), independent of time. A significant group effect indicates that the intervention influenced the outcomes compared with the control group or that there are differences between interventions. The time effect indicates whether there was a significant change over time within the studied groups (pre- and post-intervention). The interaction effect (group x time) highlights differences in changes between the groups over time. A significant interaction effect suggests that the impact of the intervention varied between groups. To compare the impact of interventions on variable changes between experimental and control groups, a two-way mixed-design ANOVA test for dependent groups with post-hoc EMM (Estimated Marginal Means) comparisons was used. Effect size (ES) was calculated using the *η*p^2^ coefficient, the ratio of the sum of squares for the effect to the sum of squares for the error:

$${\eta p}^{2}=\frac{{SS}_{effect}}{{SS}_{effect}+{SS}_{error}}$$

The interpretation of ES was as follows: 0.01 ≤ 0.05 (low effect), 0.06 ≤ 0.13 (moderate effect), and ≥0.14 (high effect) [34]. Pearson correlation coefficients (r) were used to evaluate relationships among IR, OMEN, and other measured parameters. The correlations conducted pertain to the relationships between the measured parameters in the respective groups participating in the study, taking into account the values before the start of the study and after 6 weeks of intervention, calculating the difference (delta = post-intervention value − pre-intervention value) for each variable, and then calculating the correlations. The interpretation of correlations was as follows: 0 ≤ r < 0.3, no or very weak; 0.3 ≤ r < 0.5, moderate; 0.5 ≤ r < 0.7, strong; and 0.7 ≤ r ≤ 1, very strong [35].

The minimum sample size was determined using the solve_power function from the statsmodels library in Python for ANOVA analysis: repeated measures, within-between interaction (2 measures, 3 groups). The sample size was calculated for a test power of 1-β = 0.80, *p* = 0.05, and effect size d = 0.8. Under these assumptions, the required total sample size was estimated to be 42, rounded to seven individuals per group.

The analysis was conducted according to the originally assigned groups. In all analyses, effects were considered significant if their *p*-value was less than the assumed significance level α = 0.05 (*p* < 0.05). The R programming language and tidyverse, psych, corrr, emmeans, and ggplot2 packages in the RStudio IDE were used for all calculations.

## 3. Results

Statistical analysis demonstrated a non-significant effect on group, time, and interaction on Non-Exercise Activity Thermogenesis in each of the groups that participated in this study (Table 2).

The average daily energy expenditure associated with the exercise intervention was 319.28 ± 53.53 kcal/day in group II and 299.72 ± 62.97 kcal/day in group III. No significant differences in energy expenditure during exercise were confirmed between the intervention groups (*p* = 0.53).

The two-way mixed-design analysis of variance (ANOVA) showed a significant effect of time (*p* < 0.01) and interaction (*p* < 0.01) for the BMI (Table 3). A reduction in BMI of an average of 1.3 kg/m^2^ over 6 weeks was confirmed (*p* < 0.01) in the III group. The WC decreased by 1% in the II group and by 3% in the III group, reaching significance levels (*p* < 0.01) for both time and interaction effects. Significant changes in BF and abdominal fat (ABD) content were associated with the intervention time and the interaction of time and intervention (BF%, *p* < 0.01; ABD, *p* < 0.01). Post-hoc tests indicated significant changes in the III group, showing a reduction in BF by 4.3 kg and abdominal fat by 3.8 kg after 6 weeks of intervention. The level of FFM was maintained after the 6-week intervention.

**Figure 2:**
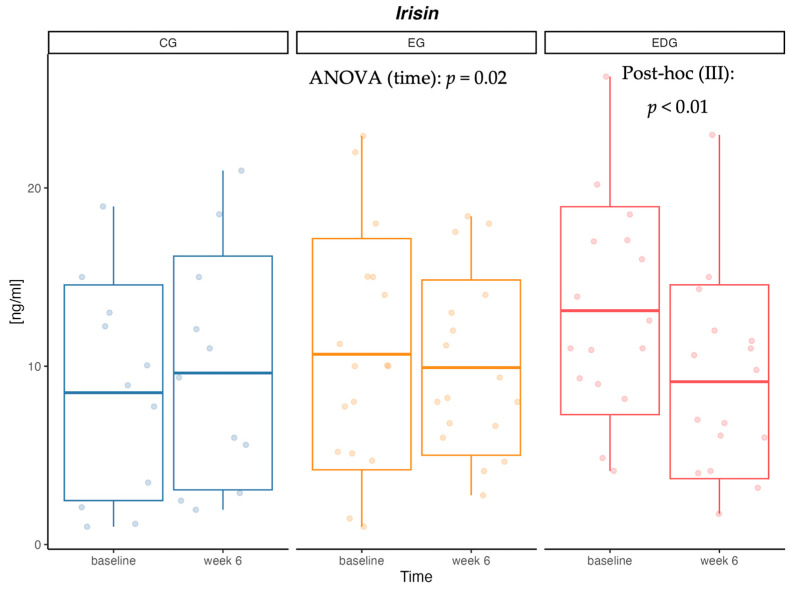
Variation in irisin (IR) [ng/mL] concentration in the control (CG I), exercise (EG II), and exercise–diet (EGD III) groups at baseline and after six weeks.

Statistical analysis demonstrated a significant effect of time (*p* = 0.02) and interaction (*p* < 0.01) on irisin (IR) concentration (Table 4, Figure 2). The change in IR concentration after 6 weeks was confirmed in the III group (*p* < 0.01). No significant differences were observed in omentin (OMEN) concentration in either the control group or the intervention groups (Table 4).

Glucose (GLU) levels did not undergo significant changes during the study (Table 4). However, there was a significant decrease in insulin (INS) levels after 6 weeks of intervention (*p* = 0.04), with a downward trend observed in both intervention groups. Post-hoc tests indicated a significant decrease in insulin (47%) in the II group (*p* < 0.01).

Low-density lipoprotein cholesterol (LDL-C) levels decreased over the six weeks (*p* = 0.02) in the intervention groups, reaching statistical significance in the III group (*p* < 0.01) (Table 4). There was also a significant interaction between time and type of intervention (*p* = 0.04), with post-hoc tests confirming a significant change in the III group (*p* < 0.01). The LDL-C level in the III group declined by 0.4 mmol/L over 6 weeks. On the other hand, no significant changes were observed in HDL-C levels (Table 4).

A change in dietary energy intake over time (*p* < 0.01), as well as the interaction between the type of intervention and time (*p* < 0.01), was indicated, and the effect size was high (*η*p^2^ = 0.13) (Table 5). Caloric intake in participants in the III group significantly declines (*p* < 0.01) by 23% per day during the intervention.

Protein intake also changed significantly among the groups (*p* = 0.04) and over time (*p* < 0.01), and the interaction between time and intervention was also significant (*p* < 0.01) (Table 5). This variability was also confirmed by a high effect size (*η*p^2^ = 0.13) for the intervention, for time (*η*p^2^ = 0.70), and the interaction (*η*p^2^ = 0.30). Participants in the III group significantly increased protein intake during the intervention (*p* < 0.01), and by the end of the 6 week intervention, protein intake was significantly higher in the III group compared with I (*p* < 0.01) and II (*p* < 0.01), although there were no significant initial differences in protein intake among groups.

Significant changes occurred in carbohydrate intake considering the interaction of time and type of intervention (*p* < 0.01). Post-hoc tests also indicated a reduction in carbohydrate intake in the III group after 6 weeks of intervention (*p* < 0.01) (Table 5).

The fiber-to-energy value of the diet intake ratio changed over time (*p* < 0.01) and in the interaction between the type of intervention and time (*p* < 0.01). The changes also confirmed the high effect size values for the intervention (*η*p^2^ = 0.12), time (*η*p^2^ = 0.07), and interaction (*η*p^2^ = 0.18). As a result of qualitative dietary changes, participants in the III group significantly changed the fiber-to-energy value of the diet over time (*p* < 0.01), which resulted in a significant difference in this ratio relative to I (*p* < 0.01) and II (*p* < 0.01) after the 6 weeks of intervention. The reduction in the fiber-to-energy value of the diet ratio in the III group was 56% after 6 weeks (Table 5).

Dietary fat levels also changed, considering the interaction of time and group (*p* < 0.01) with a moderate effect size (*η*p^2^ = 0.08). Participants in the III group reduced dietary fat intake by 4.6% (*p* < 0.01) (Table 5).

Correlations between components of body composition, adipokines, indicators of insulin resistance, and cholesterol fractions are summarized in Table 6. Overall, correlations were generally in the moderate range and overlapped for the I, II, and III groups.

Correlations between components of diet and adipokines and cholesterol fractions are summarized in Table 7. Overall, correlations were generally in the moderate range and overlapped for the I, II, and III groups.

## 4. Discussion

The effects of a 6-week aerobic-resistance exercise regimen (II group) and a combined intervention of aerobic-resistance exercise with a high-protein, low-glycemic index diet (III group) on the concentrations of irisin (IR) and omentin (OMEN), markers of carbohydrate metabolism, and cholesterol levels in men with abdominal obesity were estimated. The outcomes in the two experimental groups were compared with those in a control group (I) of men with abdominal obesity who did not receive any intervention. This study also monitored changes in body composition and selected metabolic indicators across the six-week intervention.

The intervention, which involved the implementation of both diet and exercise (III group), resulted in a significant decrease in irisin (IR) concentration by 41% and LDL-C by 14%. These effects were associated with notable changes in body composition, including a reduction in body fat (14%) and abdominal fat (31%) and the maintenance of baseline fat-free mass. The influence of exercise (II group) also resulted in beneficial changes in body composition and a significant reduction in WC, though the outcomes were considerably less than those noted in the III group. Although the trends in IR and LDL-C levels in the II group were not significant, the II group had a significant decrease in insulin concentration (47%) associated with the intervention.

The difference in outcomes was primarily due to the dietary intervention in the III group, where the use of low-glycemic index carbohydrates and increased protein intake led to an increase in fiber consumption and a reduction in dietary energy intake, resulting in a greater energy deficit than in the II group. Although not included in the presentation of results, negative correlations were noted between dietary energy value and the ratio of the fiber-to-energy value of the diet (I r = −0.71, II r = −0.61, III r = −0.78) and in the proportion of protein in the diet (I r = −0.61, II r = −0.30, III r = −0.72) in each study group. Strong negative correlations were confirmed in the III group, where recommendations for qualitative dietary changes and eating to satiety led to a significant reduction in total carbohydrate and fat intake.

The combination of aerobic and resistance exercises apparently enabled the maintenance of fat-free mass, primarily specifically skeletal muscle, in the intervention groups [36]. The III group experienced a substantial reduction in estimated fat mass over the six-week intervention. The implementation of a high-protein diet may have also prevented the utilization of skeletal muscle for energy purposes [37]. The introduction of combined resistance and aerobic exercises alone resulted in a fat loss over the six-week intervention, highlighting a synergistic effect of the combination of the dietary intervention with systematic exercise to achieve significant and meaningful changes in body composition over the relatively short interval. The changes in body composition, biochemical parameters, and adipokine levels may be closely related. Observations in the study also confirmed a significant decrease in LDL-C and IR concentrations in the III group, which combined exercise with diet; the changes in LDL-C and IR may be associated with changes in other measured parameters considered.

Patients with abdominal obesity exhibit a high cardiometabolic risk due to the presence of atherogenic dyslipidemia. The primary goal of dyslipidemia treatment is the reduction in LDL-C levels. Weight loss through diet or anti-obesity medications leads to a decrease in triglycerides (TG) and LDL-C levels and an increase in HDL-C levels [38]. In our study, the application of exercise combined with diet resulted in a reduction in LDL-C levels by 0.4 mmol/L, while HDL-C levels did not undergo significant changes. The significant reduction in LDL-C levels was associated with a substantial loss of total body fat, particularly in the visceral fat area. Other researchers have confirmed that weight reduction in obese individuals leads to a decrease in LDL-C by approximately 0.2 mmol/L (8 mg/dL) for every 10 kg of weight loss [39,40]. Our study, however, demonstrated that the combination of diet and exercise allowed for a twofold reduction in LDL-C levels with a weight loss of 3.9 kg over six weeks.

In the present study, IR concentrations significantly decreased by 4 ng/mL as a result of the combined exercise and diet intervention but by only 1.3 ng/mL in the group that engaged in exercise alone; this was not significant statistically. Serum IR concentrations tend to be significantly higher in patients with non-alcoholic fatty liver disease [41] and in patients with portal inflammation [42]. Elevated IR concentrations are observed in individuals with metabolic syndrome and insulin resistance [18,22]. However, most studies confirm higher circulating IR levels in obese patients compared with healthy individuals [43,44]. There are also variations in IR levels within the obese population, depending on coexisting metabolic disorders [45].

Although a correlation between IR and visceral obesity has been noted [45], significant correlations between IR and components of body composition were not noted in the present study, except for a moderate correlation between FFM and IR in the control group.

This relationship may be due to the fact that skeletal muscles, which are the main component of FFM, are also the primary site of IR synthesis [46]. Despite the low Pearson correlation coefficient between IR and the analyzed variables, a causal relationship and biological interactions cannot be ruled out, as there may be non-linear dependencies between adipokines, body composition, and blood biochemical parameters. The reduction in IR concentrations associated with the interventions might be attributed to the reduction in fat mass, an important site of adipokine synthesis, responsible for a significant proportion of its secretion into the blood, mainly from subcutaneous adipose tissue [19,46]. Another possible reason could be a reduction in irisin resistance, which occurs in patients with metabolic syndrome and obesity [22,43].

The present study demonstrated a correlation between IR and LDL-C concentrations after six weeks of the diet and exercise intervention. The levels of these correlated parameters declined over the interval of this study. Evidence suggests that IR is involved in cholesterol regulation, and is a crucial protein associated with communication among skeletal muscles, adipose tissue, and the liver, and also with lipid metabolism regulation [47]. IR apparently inhibits cholesterol production in the liver through an AMPK- and SREBP2-dependent mechanism [47]; the research noted a cholesterol-lowering effect by administering IR injections to obese mice over a period of two weeks.

Given the impact of IR on cholesterol metabolism and the potential presence of irisin resistance in the study sample, it is possible that with the introduction of diet and exercise, IR signaling improved. In spite of this reduction in IR levels associated with the intervention, the IR receptor in the liver may have responded more effectively, leading to a decrease in LDL cholesterol levels. A similar process occurs in insulin resistance, where interventions improve insulin signaling, reduce hormone levels, and consequently lower blood glucose levels due to enhanced insulin receptor function [48,49]. In the treatment of hypercholesterolemia, the use of statins (e.g., simvastatin) increases FNDC5 mRNA expression and IR secretion both in vitro and in vivo, highlighting the potential cholesterol-lowering effects of IR [50].

Significant correlations between IR and LDL-C concentrations and the diet of the participants were noted in the present study. Negative correlations between IR levels and the fiber-to-energy value of the diet ratio in the two intervention groups and a negative correlation between the fiber-to-energy value of the diet ratio and LDL-C in the III group underscore the substantial impact of dietary fiber on cholesterol metabolism and its related adipomyokine. The mechanism by which soluble fiber lowers cholesterol involves multiple processes. Some soluble fiber compounds bind bile acids and cholesterol during micelle formation in the enterohepatic circulation, leading to reduced cholesterol in liver cells [51]. Another area for the action of soluble fiber on cholesterol regulation is its influence on fermentation in the intestines, where the resulting metabolites can inhibit hepatic fatty acid synthesis [52]. Fiber also affects bowel movement frequency, with fiber-bound cholesterol being excreted in the stool [53]. Meta-analyses have shown that fiber intake ranging from 9 to 30 g per day, based on at least three servings, was associated with an average reduction in LDL-C of 10.6% [54]. Changes in LDL-C levels due to soluble fiber occur without significant changes in HDL cholesterol or triglyceride levels [54], as also apparent in the present study regarding HDL-C. The importance of dietary fiber and whole grains in metabolic syndrome was associated with several beneficial effects, including reduced insulin resistance, dyslipidemia, visceral obesity, and hypertension [55]. Accompanying the fiber in low-glycemic index foods, primarily vegetables and fruits, are plant sterols, which can lower LDL-C and reduce the risk of cardiovascular disease (CVD) [56].

Despite reports that high-protein diets promote de novo lipogenesis, leading to increased triglycerides and very low-density lipoprotein (VLDL) and insulin resistance [57], the present study confirmed a negative correlation between protein intake and LDL-C in the III group. The appropriate combination of exercise with a diet that increases protein intake and includes low-glycemic index carbohydrates rich in fiber may have influenced the metabolism of dietary proteins, leading to improved biochemical parameters.

Diet alone does not significantly impact IR concentrations when considering both dietary quality and energy value are considered [58]. Other studies have shown a decrease in IR concentrations caused by an energy-restricted diet (−30% energy), which was primarily related to the loss of body weight and fat [59]. Adhering to the Dietary Approaches to Stop Hypertension (DASH) diet could potentially influence IR expression, with levels linked to diet adherence [21]. The combination of aerobic and resistance exercise did not result in significant changes in IR concentrations, even after 16 weeks, in patients with obesity and metabolic syndrome [11]. However, studies combining an energy-restricted diet with exercise confirmed an increase in IR and a decrease in LDL-C after 8 weeks of intervention in the obese population [60].

Changes in omentin (OMEN) concentrations in the present study did not reach the required level of significance despite significant abdominal fat loss in the III group, the primary site of OMEN synthesis [23]. Combining aerobic and resistance exercises also did not lead to changes in OMEN concentrations, even after 16 weeks [61]. The significant weight loss results in the present study were observed in the group subjected to exercise and diet, but the lack of significant changes in OMEN concentrations could be due to the relatively short intervention period of 6 weeks. Extending the intervention period with the applied procedures, i.e., exercise and diet, might lead to increased OMEN concentrations [25]. However, correlations between IR and OMEN were noted in the intervention groups. Negative correlations between OMEN and LDL-C in the I and II groups were also noted. The effect of increased OMEN concentrations was observed in a dietary intervention study including a low-energy diet (500–1000 kcal daily deficit) for 4 months. The intervention resulted in 13.8% weight loss and a 22.1% increase in OMEN concentrations, though a correlation between OMEN and LDL was not observed [25].

The present study has several limitations. The duration of the intervention was relatively short and amounted to six weeks. A notable limitation is the absence of a group to evaluate the influence of diet alone on the analyzed parameters. Additionally, the VO2 max test was not conducted during the project, which would have allowed for monitoring training adaptations in the intervention groups. The study also does not fully elucidate the complex mechanisms underlying changes in adipokine concentrations following exercise and dietary interventions.

## 5. Conclusions

In summary, the results of the present study demonstrated that combining exercise with dietary intervention leads to a reduction in irisin (IR) concentrations and reduces abdominal obesity. These effects were associated with notable changes in body composition, including a reduction in body fat and abdominal fat and the maintenance of baseline fat-free mass. The difference in outcomes was primarily due to the dietary intervention in the III group, where the use of low-glycemic index carbohydrates and increased protein intake led to an increase in fiber consumption and a reduction in dietary energy intake, resulting in a greater energy deficit than in the II group. The reduction in IR concentrations associated with the intervention can, therefore, be attributed to a reduction in fat mass, an important site of adipokine synthesis, responsible for a significant part of their secretion into the blood. Changes observed between LDL-C and IR concentrations may be interrelated and also attributable to dietary fiber intake. The implementation of aerobic-resistance exercise alone positively affects body composition and leads to a reduction in insulin levels but does not influence changes in LDL-C, IR, or OMEN concentrations. Overall, the findings highlight the effect of combining dietary and exercise interventions to achieve significant changes in body composition, even over a relatively short interval.

## Figures and Tables

**Table 1:** Characteristics of the research participants in the control (I), exercise (II), and exercise–diet (III) groups.

Index	Group			*p*-Value
	I	II	III	
Age [years]	34.1 ± 5.5	34.8 ± 6.0	34.9 ± 5.6	0.92
WC [cm]	111.2 ± 6.8	110.8 ± 11.3	109.0 ± 7.6	0.75
BM [kg]	101.9 ± 11.5	105.6 ± 7.4	105.6 ± 10.3	0.40
BF [kg]	36.2 ± 6.6	36.8 ± 9.0	36.0 ± 6.0	0.13
ABD [kg]	16.6 ± 4.5	16.5 ± 6.4	16.1 ± 4.2	0.55
FFM [kg]	69.8 ± 8.7	70. 6 ± 8.2	70.1 ± 7.2	0.68

I—control group, II—exercises group, III—exercises combined with diet group, WC—waist circumference, BM—body mass, BF— body fat, ABD—abdominal fat, FFM—fat-free mass, *p* < 0.05—statistically significant difference.

**Table 2:** Non-Exercise Activity Thermogenesis (NEAT) in the control (I), exercise (II), and exercise–diet (III) groups at baseline and after six weeks, and results of statistical comparisons among groups.

Variable	Time	Groups Mean (SD)			Effect	*F*	*p*	*η*p^2^	Post-Hoc Test, *p*					
		I	II	III					Baseline			Week 6		
NEAT [kcal/ day]	baseline	521.80 (188.24)	480.05 (95.28)	580.56 (97.52)	group	1.69	0.20	0.07	-	-	-	-	-	-
	week 6	518.99 (176.38)	522.16 (109.41)	574.83 (102.26)	time	0.50	0.48	0.01	-	-	-	-	-	-
					interaction	1.01	0.38	0.01	-	-	-	-	-	-

SD—standard deviation, *p* < 0.05—statistically significant difference, *η*p^2^—effect size.

**Table 3:** Body composition: body mass index (BMI), waist circumference (WC), body fat (BF), abdominal fat (ABD), and fat-free mass (FFM) in the control (I), exercise (II), and exercise–diet (III) groups at baseline and after six weeks, and results of statistical comparisons among groups.

Variable	Time	Groups Mean (SD)			Effect	*F*	*p*	*η*p^2^	Post-Hoc Test *, p					
		I	II	III					Baseline			Week 6		
BMI [kg/ m^2^]	baseline	31.7 (3.7)	32.2 (4.3)	32.1 (3.7)	group	0.14	0.87	0.01	-	-	-	-	-	-
	week 6	31.9 (3.7)	32.1 (4.4)	30.8 (3.5)	time	15.10	<0.01	0.01	-	-	-	I: NS	II: NS	III: <0.01
					interaction	18.92	<0.01	0.01	I: NS	II: NS	III: NS	I: NS	II: NS	III: <0.01
WC [cm]	baseline	111.2 (6.8)	110.8 (11.3)	109.0 (7.6)	group	0.83	0.44	0.04	-	-	-	-	-	-
	week 6	112.0 (6.3)	109.7 (10.9	105.9 (7.4)	time	13.28	<0.01	0.01	-	-	-	I: NS	II: 0.02	III: <0.01
					interaction	12.54	<0.01	0.01	I: NS	II: NS	III: NS	I: NS	II: NS	III: <0.01
BF [kg]	baseline	36.2 (6.6)	36.8 (9.0)	36.0 (6.0)	group	0.59	0.56	0.03	-	-	-	-	-	-
	week 6	36.3 (6.5)	35.9 (9.0)	31.7 (5.0)	time	36.35	<0.01	0.02	-	-	-	I: NS	II: NS	III: <0.01
					interaction	23.70	<0.01	0.02	I: NS	II: NS	III: NS	I: NS	II: NS	III: <0.01
ABD [kg]	baseline	16.6 (4.5)	16.5 (6.4)	16.1 (4.2)	group	0.91	0.47	0.03	-	-	-	-	-	-
	week 6	16.5 (4.6)	15.8 (6.5)	12.3 3.5	time	44.84	<0.01	0.02	-	-	-	I: NS	II: NS	III: <0.01
					interaction	27.67	<0.01	0.03	I: NS	II: NS	III: NS	I: NS	II: NS	III: <0.01
FFM [kg]	baseline	66.0 (6.8)	68.6 (10.2)	70.1 (7.2)	group	0.77	0.47	0.04	-	-	-	-	-	-
	week 6	65.9 (7.0)	68.5 (9.9)	69.6 (7.4)	time	0.88	0.35	0.01	-	-	-	-	-	-
					interaction	0.25	0.78	0.01	-	-	-	-	-	-

SD—standard deviation, *p* < 0.05—statistically significant difference, NS—not significant, *η*p^2^—effect size, * row 1: effect of group, between groups; row 2: effect of time, within group; row 3: effect of group-time interaction.

**Table 4:** Concentrations of irisin (IR), omentin (OMEN), glucose (GLU), insulin (INS), low-density lipoprotein cholesterol (LDL-C), and high-density lipoprotein cholesterol (HDL-C) in the control (I), exercise (II) and exercise–diet (III) groups at baseline and after six weeks, and results of statistical comparisons among groups.

Variable	Time	Groups Mean (SD)			Effect	*F*	*p*	*η*p^2^	Post-Hoc Test *, p					
		I	II	III					Baseline			Week 6		
IR [ng/ mL]	baseline	8.5 (6.0)	11.3 (6.2)	13.1 (5.8)	group	0.47	0.63	0.02	-	-	-	-	-	-
	week 6	9.6 (6.6)	10.0 (5.1)	9.1 (5.4)	time	5.90	0.02	0.01	-	-	-	I: NS	II: NS	III: <0.01
					interaction	6.50	<0.01	0.03	I: NS	II: NS	III: NS	I: NS	II: NS	III: <0.01
OMEN [ng/ mL]	baseline	350.1 (196.8)	281.3 (260.8)	338.9 (205.7)	group	0.54	0.58	0.02	-	-	-	-	-	-
	week 6	346.7 (186.3)	258.3 (254.3)	325.6 (219.1)	time	0.30	0.58	0.01	-	-	-	-	-	-
					interaction	0.05	0.95	0.01	-	-	-	-	-	-
GLU [mmol/L]	baseline	5.1 (0.6)	5.2 (0.3)	5.4 (1.8)	group	0.15	0.86	0.01	-	-	-	-	-	-
	week 6	5.0 (0.3)	5.0 (0.4)	5.1 (1.0)	time	3.15	0.08	0.01	-	-	-	-	-	-
					interaction	0.18	0.84	0.01	-	-	-	-	-	-
INS [μIU/mL]	baseline	15.2 (6.2)	18.5 (10.2)	14.2 (8.4)	group	1.48	0.24	0.04	-	-	-	-	-	-
	week 6	16.3 (7.5)	12.6 (5.4)	10.7 (4.2)	time	4.32	0.04	0.04	-	-	-	I: NS	II: <0.01	III: NS
					interaction	2.20	0.12	0.04	-	-	-	-	-	-
LDL-C [mmol/L]	baseline	3.4 (0.8)	3.2 (1.1)	3.2 (0.8)	group	0.62	0.54	0.03	-	-	-	-	-	-
	week 6	3.4 (0.8)	3.0 (0.8)	2.8 (0.6)	time	6.38	0.02	0.02	-	-	-	I: NS	II: NS	III: <0.01
					interaction	3.70	0.04	0.02	I: NS	II: NS	III: NS	I: NS	II: NS	III: <0.01
HDL-C [mmol/L]	baseline	1.1 (0.2)	1.2 (0.3)	1.3 (0.4)	group	0.30	0.74	0.01	-	-	-	-	-	-
	week 6	1.2 (0.2)	1.2 (0.3)	1.2 (0.3)	time	0.03	0.87	0.01	-	-	-	-	-	-
					interaction	2.42	0.10	0.01	-	-	-	-	-	-

SD—standard deviation, *p* < 0.05—statistically significant difference, NS—not significant, *η*p^2^—effect size, * row 1: effect of group, between groups; row 2: effect of time, within group; row 3: effect of group-time interaction.

**Table 5:** Energy value of the diet, and the percentage of total energy intake of proteins, carbohydrates, the fiber-to-energy value of the diet index, and fats of participants’ diet in the control (I), exercise (II), and exercise–diet (III) groups at baseline and after six weeks, and results of statistical comparisons among groups.

Variable	Time	Groups Mean (SD)			Effect	*F*	*p*	*η*p^2^	Post-Hoc Test *, p					
		I	II	III					Baseline			Week 6		
Energy value of the diet [kJ]	baseline	11,212.2 (1431.8)	11,505.5 (1716.4)	12,134.3 (1832.2)	group	0.62	0.54	0.03	-	-	-	-	-	-
	week 6	11,588.4 (1451.0)	11,743.7 (1834.8)	9891.4 (1404.9)	time	8.72	<0.01	0.03	-	-	-	I: NS	II: NS	III: <0.01
					interaction	23.0	<0.01	0.13	I: NS	II: NS	III: NS	I: NS	II: NS	III: <0.01
Proteins [%]	baseline	21.4 (5.1)	18.6 (3.6)	16.9 (2.8)	group	3.38	0.04	0.13	I: NS	II: NS	III: NS	I/II: NS	I/III: <0.01	III/II: 0.01
	week 6	19.8 (4.1)	17.6 (3.3)	25.9 (4.5)	time	17.4	<0.01	0.07	-	-	-	I: NS	II: NS	III: <0.01
					interaction	48.3	<0.01	0.30	I: NS	II: NS	III: NS	I: NS	II: NS	III: <0.01
Carbohydrates [%]	baseline	45.7 (8.0)	46.4 (6.6)	46.4 (6.6)	group	1.93	0.16	0.07	-	-	-	-	-	-
	week 6	43.3 (7.4)	49.5 (5.5)	42.0 (5.3)	time	1.51	0.23	0.01	-	-	-	-	-	-
					interaction	5.30	<0.01	0.06	I: NS	II: NS	III: NS	I: NS	II: NS	III: <0.01
Fiber/energy value of the diet [mJ]	baseline	2.2 (0.95)	2.1 (0.6)	1.9 (0.8)	group	3.05	0.04	0.12	I: NS	II: NS	III: NS	I/II: NS	I/III: <0.01	III/II: 0.01
	week 6	2.1 (1.0)	2.1 (0.7)	3.4 (0.8)	time	22.74	<0.01	0.07	-	-	-	I: NS	II: NS	III: <0.01
					interaction	32.67	<0.01	0.18	I: NS	II: NS	III: NS	I: NS	II: NS	III: <0.01
Fats [%]	baseline	33.1 (7.9)	34.8 (5.5)	36.7 (6.8)	group	0.13	0.88	0.01	-	-	-	-	-	-
	week 6	36.8 (5.4)	33.0 (4.8)	32.1 (5.1)	time	0.74	0.40	0.01	-	-	-	-	-	-
					interaction	5.25	<0.01	0.08	I: NS	II: NS	III: NS	I: NS	II: NS	III: <0.01

SD—standard deviation, *p* < 0.05—statistically significant difference, NS—not significant, *η*p^2^—effect size, * row 1: effect of group, between groups; row 2: effect of time, within group; row 3: effect of group-time interaction.

**Table 6:** Correlations between components of body composition and adipokines, indicators of insulin resistance, and cholesterol fractions in the control (I), exercise (II), and exercise–diet (III) groups †.

	BMI [kg/m^2^]	WC [cm]	BF [kg]	ABD [kg]	FFM [kg]	IR [ng/mL]	OMEN [ng/mL]	GLU [mmol/L]	INS [μIU/mL]	LDL-C [mmol/L]	HDL-C [mmol/L]
IR I [ng/mL]	0.16	0.04	0.17	0.09	0.60 *	-	0.20	−0.31 *	−0.44 *	0.17	−0.28
IR II [ng/mL]	−0.12	−0.06	−0.22	−0.20	0.02	-	0.56 *	0.09	−0.12	0.11	0.08
IR III [ng/mL]	−0.16	−0.15	−0.11	−0.01	−0.07	-	0.65 *	−0.13	−0.13	0.30 *	0.04
OMEN I [ng/mL]	0.02	−0.08	−0.04	−0.02	−0.20	0.20	-	0.10	−0.05	−0.31 *	0.10
OMEN II [ng/mL]	0.06	0.13	0.11	0.11	0.15	0.56 *	-	0.19	0.00	−0.30 *	0.20
OMEN III [ng/mL]	−0.07	−0.04	−0.15	−0.06	−0.11	0.65 *	-	−0.09	−0.07	−0.06	0.20
LDL-C I [mmol/L]	−0.40 *	−0.05	0.09	0.25 *	−0.32 *	0.17	−0.31 *	−0.23 *	0.12	-	0.35 *
LDL-C II [mmol/L]	−0.28	−0.26	−0.32 *	−0.30 *	−0.49 *	0.11	−0.30 *	−0.34 *	−0.06	-	−0.18
LDL-C III [mmol/L]	−0.01	0.00	0.32 *	0.34 *	−0.07	0.24 *	−0.06	0.10	0.20	-	−0.02

*—statistically significant value *p* < 0.05; † Concentrations of irisin (IR), omentin (OMEN), glucose (GLU), insulin (INS), low-density lipoprotein cholesterol (LDL-C), and high-density lipoprotein cholesterol (HDL-C), body mass index (BMI), waist circumference (WC), body fat (BF), abdominal fat (ABD) and fat-free mass (FFM) in each of the three groups (I, II, III) are based on the two-time points.

**Table 7:** The value of the Pearson correlation for adipokines, cholesterol fractions, and diet components in the control (I), exercise (II), and exercise–diet (III) groups †.

	Energy Value of the Diet [kJ]	Proteins [%]	Carbohydrates [%]	Fiber/Energy Value of the Diet [mJ]	Fats [%]
IR I [ng/mL]	−0.07	−0.22	0.52 *	−0.23	−0.44 *
IR II [ng/mL]	0.31	−0.14	−0.15	−0.55 *	0.27
IR III [ng/mL]	0.32 *	−0.18	0.32 *	−0.38 *	−0.15
OMEN I [ng/mL]	−0.52 *	−0.13	−0.12	0.06	0.22
OMEN II [ng/mL]	0.40 *	−0.21	−0.03	−0.29	0.17
OMEN III [ng/mL]	0.06	0.09	−0.08	−0.22	0.00
LDL-C I [mmol/L]	−0.52 *	0.23	−0.39 *	0.01	0.36 *
LDL-C II [mmol/L]	−0.20	0.21	−0.08	−0.18	−0.06
LDL-C III [mmol/L]	0.45 *	−0.41 *	0.36 *	−0.61 *	0.03

*—statistically significant value *p* < 0.05; † Concentrations of irisin (IR), omentin (OMEN), and low-density lipoprotein cholesterol (LDL-C) in each of the three groups (I, II, III) are based on the two-time points.

## Data Availability

Data are available on request from the corresponding author.

## Supplementary Materials

The following supporting information can be downloaded at: https://www.mdpi.com/article/10.3390/nu16203480/s1. Table S1. A general strategy for a combined aerobic and resistance training program intended for groups engaged in aerobic–resistance exercises (II and III).
