# Supplementary material for: Positive Effects of Aerobic-Resistance Exercise and an Ad Libitum High-Protein, Low-Glycemic Index Diet on Irisin, Omentin, and Dyslipidemia in Men with Abdominal Obesity: A Randomized Controlled Trial
Source: Nutrients. 2024 Oct 14;16(20):3480. doi: 10.3390/nu16203480 (PMC11510197; doi:10.3390/nu16203480)
Supplement: Supplementary file 1 [file nutrients-16-03480-s001.zip › nutrients-3190742-supplementary.pdf]

Table S1. A general strategy for a combined aerobic and resistance training program intended for a groups engaged in aerobic–resistance exercises (EG and EDG).

	**Training A**	**Training B**
Duration of aerobic training [min]	10	10
Intensity of aerobic [% HR max]	70	70
Duration of resistance training [min]	40	40
Intensity of resistance training [% 1 RM]	70	70
Volume of resistance training [exercises x series x repetitions]	6 x 4 x 12	6 x 4 x 12
Breaks between series [min]	1	1
Type of training	Push	Pull
Specialized exercises	1. Supported push-up<br>2. Barbell bench press<br>3. Standing dumbbell press<br>4. Cable triceps extension<br>5. Front squats with kettlebell<br>6. Plank	1. One arm row dumbbell<br>2. Reverse grip lat pulldown<br>3. Bent dumbbell row<br>4. Standing dumbbell curl<br>5. Dumbbell deadlift<br>6. Hip thrust lying

HR max—maximal heart rate, 1RM—one repetition maximum.

Following an aerobic warm-up at 70% of HR max, the resistance training system consisted of alternating between two exercise sets: Training A - "push" and Training B - "pull." The exercise sequence in the "push" set allowed for the initial engagement of the chest muscles (barbell bench press, supported push-ups), followed by the shoulder muscles (standing dumbbell press), triceps muscles (cable triceps extension), lower limb muscles performing squats (front squats with kettlebell), concluding with the activation of abdominal muscles (plank). The "pull" training commenced with the involvement of the back muscles in the first 3 exercises (one-arm row with dumbbell, reverse grip lat pulldown, bent dumbbell row), followed by isolated exercises targeting the biceps (standing dumbbell curl), concluding with exercises targeting the hamstrings and gluteal muscles (dumbbell deadlift, hip thrust lying). After the resistance exercises, study participants entered a cooling-down phase by reducing the intensity to 50% of HR max for one minute. The exercise session concluded with breathing exercises and stretching of the engaged muscle groups.
